# The Therapeutic Effects of Semaglutide in Congenital Linear Scleroderma

**DOI:** 10.7759/cureus.88725

**Published:** 2025-07-25

**Authors:** Helen Chen, Mary Elhawi, Michelle Tarbox

**Affiliations:** 1 Department of Dermatology, Texas Tech University Health Sciences Center, Lubbock, USA

**Keywords:** congenital linear scleroderma, connective tissue disease, glp-1 agonists, linear scleroderma, semaglutide

## Abstract

Congenital linear scleroderma (CLS) is a rare connective tissue disorder characterized by unilateral linear induration that can lead to restricted mobility and limb deformities. Various treatments have been proposed, including D-penicillamine, phototherapy, oral vitamin D, and immunosuppressants such as methotrexate (MTX), cyclosporine, and interleukin-6 (IL-6) inhibitors. We present the case of a 14-year-old female patient with refractory CLS, whose condition continued to worsen despite treatment with tocilizumab, mycophenolate mofetil (MMF), and MTX. She was subsequently started on a glucagon-like peptide-1 (GLP-1) receptor agonist and began to experience improved mobility in her left arm and decreased skin hardening. To our knowledge, this is the first reported case suggesting the potential role of GLP-1 receptor agonists in slowing the progression of localized scleroderma, possibly due to their anti-fibrotic and anti-inflammatory effects.

## Introduction

Congenital linear scleroderma (CLS) is a rare inflammatory connective tissue disease that leads to skin and subcutaneous tissue sclerosis that usually presents at birth. There are two types of sclerodermas: juvenile systemic sclerosis (JSS), characterized by cutaneous and visceral involvement, and juvenile localized scleroderma (JLS), limited to the skin and underlying tissues [1]. The main subtypes of JLS include plaque morphea, linear scleroderma, mixed morphea, and pansclerotic morphea. CLS presents as unilateral linear cutaneous indurations on the face, scalp, or extremities that can lead to restrictive mobility and limb deformities. Proposed treatments include D-penicillamine, topical or oral vitamin D, phototherapy, phenytoin, corticosteroids, methotrexate (MTX), mycophenolate mofetil (MMF), cyclosporine, and interferons [1,2].

## Case presentation

A 14-year-old female patient with a history of CLS presented for dermatologic evaluation. She had been under routine care by pediatric rheumatology every three months for disease management. She was diagnosed with CLS at the age of six and initially treated with solumedrol pulse dosing, MTX, and low-dose oral steroids for over a year, which stabilized lesions on her left arm. However, five months after discontinuing steroids, she returned with a decreased range of motion in her left thumb and increasing pain in her left arm, prompting the initiation of MMF.

**Figure 1:**
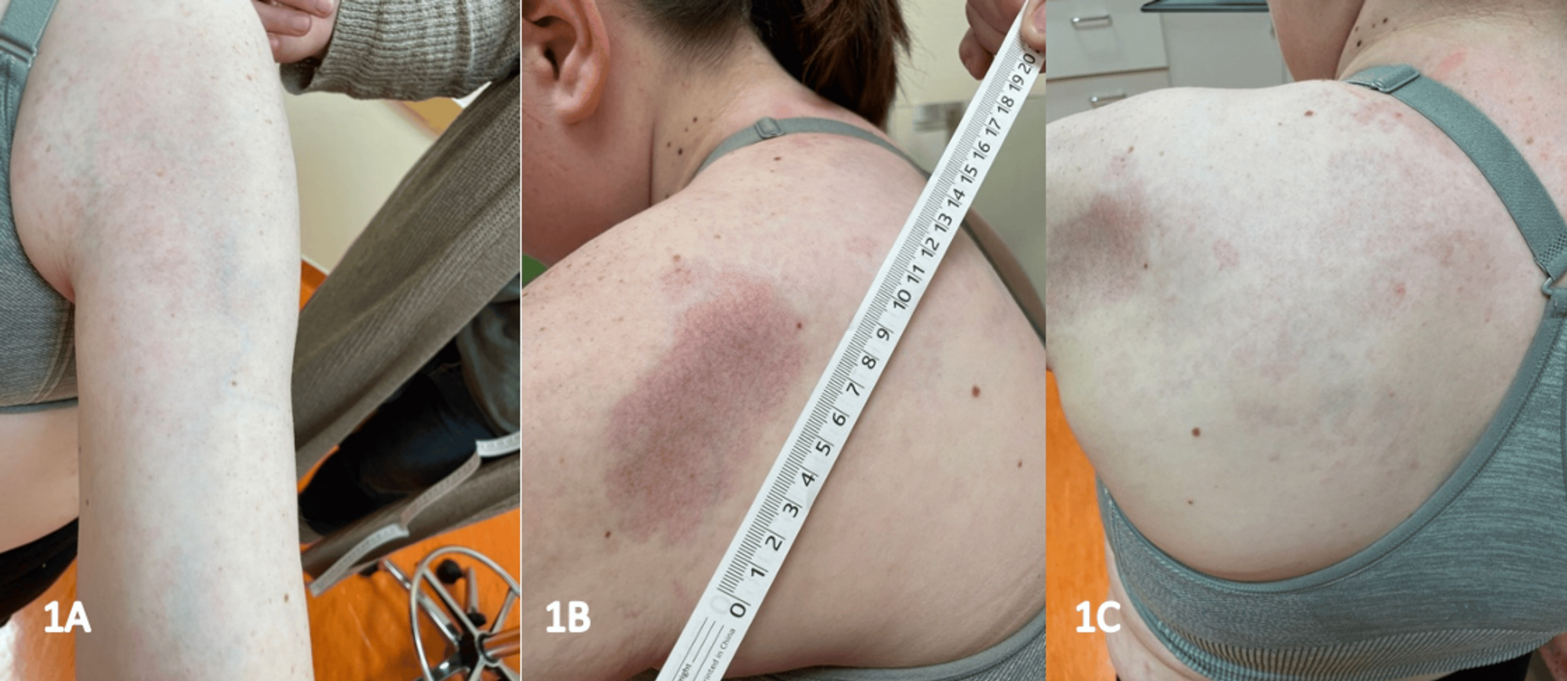
Clinical photos show a 7.8 × 3.7 cm erythematous atrophic plaque on the left posterior shoulder, along with generalized erythema on the left upper back. Mild erythema and worsening sclerosis were noted on the left upper forearm, with warmth on palpation. The active findings on the posterior left shoulder and left biceps prompted initiation of tocilizumab, while continuing her current doses of mycophenolate 1000 mg twice daily and methotrexate 20 mg weekly

Over the following years, she experienced intermittent disease flares requiring increasing dose adjustments of MTX and MMF, which helped maintain stable lesions on magnetic resonance imaging (MRI). However, in the year leading up to her dermatology visit, she developed worsening erythema, atrophy of the left shoulder and scapular region, and progressive thinning of the subcutaneous fatty tissue, as confirmed on MRI. She was initially prescribed abatacept, but due to financial constraints, she was ultimately started on tocilizumab 162 mg every other week, in combination with MMF 1000 mg BID and MTX 20 mg weekly (Figure 1). Despite four months of this regimen, she continued to experience progressive skin hardening and restricted mobility in her left arm.

**Figure 2:**
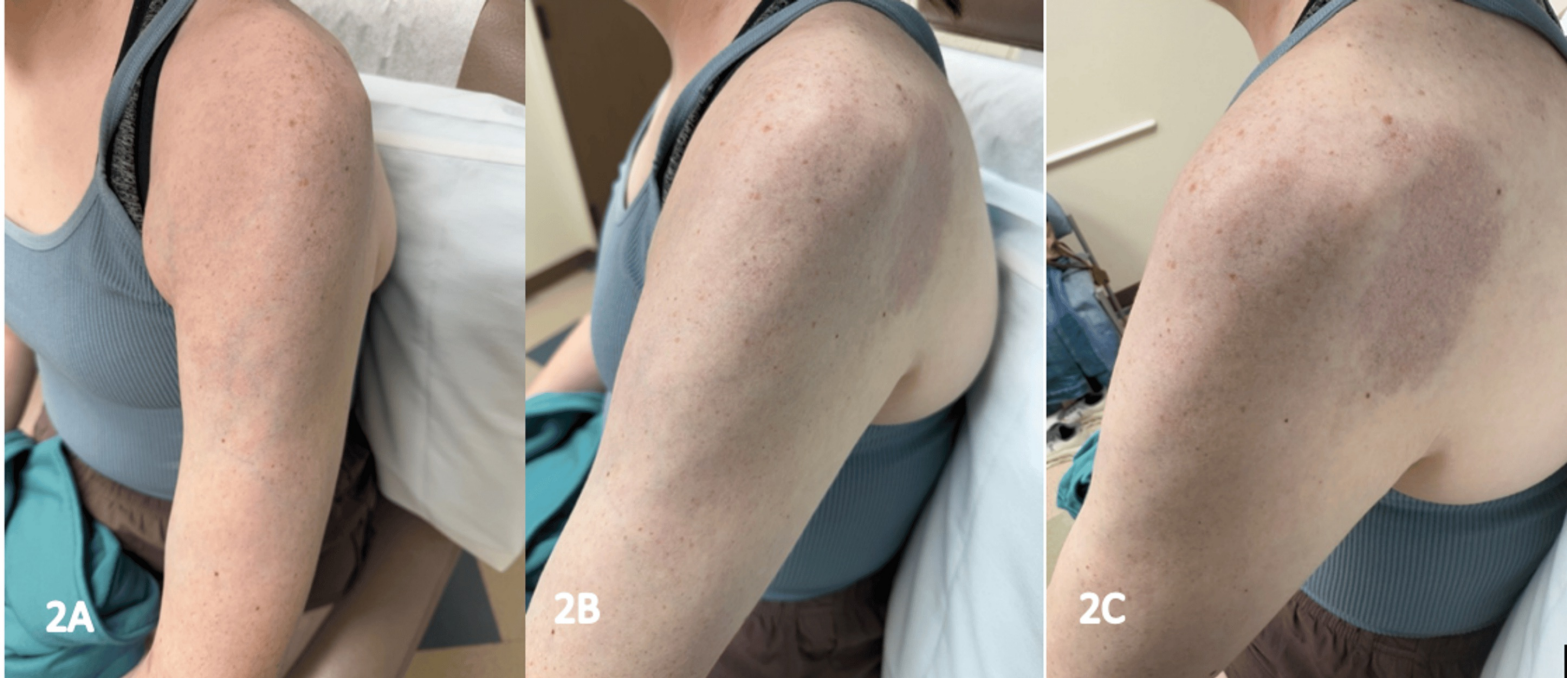
Clinical photos depict mild atrophy of the left upper arm and posterior shoulder, accompanied by a faint purple-to-pink hue. On physical examination, the skin was supple and mobile to palpation. These findings were observed five months after initiating semaglutide

Unrelated to her CLS therapy, the patient was started on semaglutide due to weight gain likely associated with prolonged steroid use. Shortly after initiating semaglutide, she reported improved mobility in her left arm and reduced skin hardness, suggesting a potential therapeutic effect. At her dermatology visit seven months after starting semaglutide, she had not experienced any worsening or flares of her scleroderma, and she felt that her left arm continued to have improved mobility. On physical exam, there was mild atrophy of her left arm with a faint purple-to-pink hue on the upper arm and left posterior shoulder (Figure 2). Her skin was supple on palpation, and no pronounced erythema was noted.

## Discussion

CLS is a subtype of JLS characterized by one or more linear streaks of cutaneous induration that involve the dermis, subcutaneous tissue, muscle, and sometimes underlying bone [3]. Diagnosis is often delayed, with an average lag of four years, a delay that our patient experienced. Initially incorrectly diagnosed with subcutaneous fat necrosis in infancy, she was officially diagnosed with CLS at six years old. CLS typically presents unilaterally on the trunk, extremities, and more commonly, on the face, with facial involvement occurring in 66% of cases, often manifesting as an en coup de sabre appearance [3].

Diagnosing scleroderma in the early inflammatory phase can be challenging as it lacks the classic histologic features. During this stage, dense, homogenized, eosinophilic collagen surrounds adnexal structures and vessels, accompanied by perivascular and periadnexal lymphohistiocytic inflammation [4]. The dermis and hypodermis may display a tumefied endothelium with a decreased lumen [5]. As the disease progresses, inflammation subsides, leading to intense dermal and sometimes subcutaneous sclerosis with trapping of adnexal structures, characteristic of scleroderma/morphea. Clinically, this presents as skin surface depression and hardening, reflecting the loss of adipose tissue replaced by sclerotic collagen.

Due to the rarity of JLS, including its congenital linear subtype, multiple European pediatric rheumatologists released a consensus-based recommendation in 2019 [2]. This consensus emphasized the importance of multidisciplinary care involving pediatric rheumatology and ophthalmology, as well as the use of MRI for assessing musculoskeletal or neurological involvement [2]. Early treatment initiation is advised to reduce morbidity and prevent limb deformities.

While there is currently no agreed-upon treatment for CLS, various therapeutic approaches have been suggested, including D-penicillamine, topical or oral vitamin D, phototherapy, phenytoin, corticosteroids, MTX, cyclosporine, and interferons [4]. The consensus statement recommended starting systemic corticosteroids for the active inflammatory phase of severe JLS, particularly in linear, generalized, or pansclerotic subtypes, along with MTX or an alternative disease-modifying anti-rheumatic drug (DMARD) [2]. For patients with persistent flares, MMF was advised as an add-on or alternative therapy [2].

UVA1 phototherapy, which penetrates deeper into the dermis than ultraviolet B (UVB) or psoralen ultraviolet A (PUVA), is a safe and effective treatment for all forms of localized scleroderma in adults [6]. Large studies in the pediatric population are lacking. Due to the unknown potential long-term risk of carcinogenesis, clinicians must conduct a thorough risk-benefit analysis before considering phototherapy in children, and the optimal dosing and maintenance regimen remain unclear.

Various mechanisms of action have been proposed for scleroderma, particularly the role of innate and adaptive immune systems in upregulating profibrotic and proinflammatory cytokines, such as transforming growth factor beta (TGF-β) and interleukin-6 (IL-6), respectively [2]. Glucagon-like peptide-1 (GLP-1) receptor analogs have been shown to inhibit inflammatory and immune responses by reducing cytokines, like IL-6, interferon gamma (IFN-γ), IL-17, and IL-2 while upregulating IL-10, which promotes an anti-inflammatory phenotype in cultured human macrophages [7]. The reduction of renal fibrosis with the GLP-1 analog liraglutide is hypothesized to result from the inhibition of TGF-β activation [8]. These anti-inflammatory and anti-fibrotic effects have also been observed in metabolic conditions, particularly cardiovascular disease [9].

The potential anti-inflammatory and anti-fibrotic properties of GLP-1 agonists raise the question of whether medications in this class, such as semaglutide, could help mitigate the progression of localized scleroderma. GLP-1 agonists may have utility in the treatment of children with CLS, and we advocate for larger studies to evaluate the role of GLP-1 in treating inflammatory connective tissue diseases.

## Conclusions

This case highlights a potential therapeutic role for GLP-1 receptor agonists in CLS, a rare and challenging subtype of JLS. Our patient showed clinical improvement in mobility and skin softening following initiation of semaglutide, despite prior failure of conventional immunosuppressive therapies. Given the known anti-inflammatory and anti-fibrotic properties of GLP-1 agonists, further studies are warranted to investigate their role in treating inflammatory connective tissue diseases.
